# Diseases of Summer Time

**Published:** 1875-08

**Authors:** E. H. Gibb


### DISEASES OF SUMMER TIME.

BY E. H. GIBB, M.D.

The seeds of disease are everywhere; they only require for their germination and development those conditions which individuals and communities are always supplying, by their criminal indifferance to sanitary laws. If we make a tour of inspection through the tenement house districts of any city, we shall fail to see those evidences of advancement of which civilization boasts. We cannot imagine a state of society, ever so primitive, that would suffer in comparison with that which so horrifies and disgusts us.

These are the great nursery beds of pestilence that walketh in darkness, the homes of reeking vice and crime. From these central points, contamination spreads in all directions. We must remember that "cleanliness is next to godliness." Without it there can be neither bodily or spiritual health. Scrupulous neatness of person is not sufficient to ward off disease. Our homes and their surroundings must be as free from the germs of disease as it is possible to make them. This will be found a difficult task, for our enemies are legion. We can have our surroundings in complete order, but we cannot always control others.

Make a home perfect in all its appointments; fulfill every sanitary condition as to cleanliness, ventilation and drainage. Let food and raiment be selected with the greatest care, let life be free from irregularities and excesses, and even then, on some fine summer day, some neighbor will open Pandora's box, for the escape of the demons of disease.

It is one of the penalties we pay for the many privileges of society. We are compelled to accept the present condition of things with the hope we may improve it.

The summer months should be the healthiest season of the year. The temperature is agreeable to the conditions of health, and changes are not sudden and extreme. The air is kept pure and wholesome by showers and the action of electricity. That it is the most sickly season of the year, is the fault of men.

The heat induces the fermentation and decay of organic matters and the commingling with the atmosphere of the noxious gases thus generated. These are the seeds of the various diseases that are so destructive during the summer. It is comparatively but a short time since the origin of these diseases was discovered, although it was long suspected. When the history, the habits and the course of the enemy is understood, it should be possible to avoid him. Fortunately, that is true in this case. If we do not destroy the seeds of disease by sanitary means, we can fortify ourselves against attack, or render an attack almost harmless by proper attention to our mode of living. I would rank personal cleanliness above all things. It comprehends a great deal. A person can not be cleanly who uses tobacco in any form, or who uses liquor as a beverage, or who wears clothing soiled, so as to contaminate the air breathed. I never have had much faith in what are called "regular habits" as the term is generally understood. It has been the scape-goat for untold misery. There is no reason why a man should rise at six o'clock to-morrow morning, to suffer all day, and to a certain extent all his life, for the want of sixty minutes more sleep. The fact that he has been in the *habit* of rising at six is no reason. The conditions may have been different. To dine at one o'clock, because that is the dinner hour, though you are not hungry, is gluttony, pure and simple.

To suffer the pangs of hunger till the senseless clock points to the "regular supper hour" is a direct invitation to the horrors of dyspepsia. If we would escape disease we must keep the system "toned up." Eat plenty of wholesome, properly cooked food, *when the stomach craves it.* We must get enough sleep and rest to thoroughly refresh us, and at such times as nature demands. Nature in respect to food and sleep, always dictates.

It is the refinement of cruelty to treat little children as they are often treated through mistaken kindness. A child should have food whenever it manifests a desire for it, no matter what the hour of day or night. Its cravings are more unerring than our judgment. A fretful infant will be quieted by a little food or a drink of cold water, three times in four. Thousands of parents are to-day mourning over the loss of their little ones, starved to death through ignorance.

To secure good health and long life, we have simply to be governed by reason and common sense. A simple rule that covers the whole ground is, to keep ourselves comfortable.

